# Mechanism of increased risk of insulin resistance in aging skeletal muscle

**DOI:** 10.1186/s13098-020-0523-x

**Published:** 2020-02-11

**Authors:** Jian Shou, Pei-Jie Chen, Wei-Hua Xiao

**Affiliations:** School of Kinesiology, Shanghai University of Sport, 200 Hengren Road, Yangpu District, Shanghai, 200438 China

**Keywords:** Skeletal muscle aging, Insulin resistance, Mechanism

## Abstract

As age increases, the risk of developing type 2 diabetes increases, which is associated with senile skeletal muscle dysfunction. During skeletal muscle aging, mitochondrial dysfunction, intramyocellular lipid accumulation, increased inflammation, oxidative stress, modified activity of insulin sensitivity regulatory enzymes, endoplasmic reticulum stress, decreased autophagy, sarcopenia and over-activated renin-angiotensin system may occur. These changes can impair skeletal muscle insulin sensitivity and increase the risk of insulin resistance and type 2 diabetes during skeletal muscle aging. This review of the mechanism of the increased risk of insulin resistance during skeletal muscle aging will provide a more comprehensive explanation for the increased incidence of type 2 diabetes in elderly individuals, and will also provide a more comprehensive perspective for the prevention and treatment of type 2 diabetes in elderly populations.
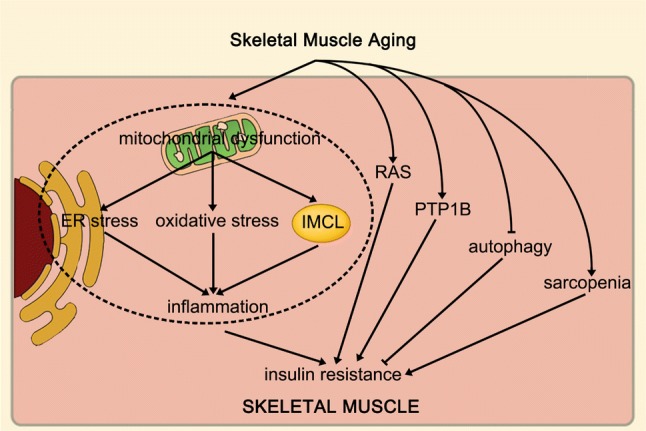

## Background

Globally, the incidence of diabetes and the number of patients with diabetes are rising sharply, which has resulted in a worldwide public health problem that seriously threatens human health. According to the international diabetes federation (IDF), there are nearly 500 million people with diabetes in the world, and approximately 80% of people with diabetes live in low- income and middle-income countries. In 2017, there were approximately 425 million people with diabetes in the world between the ages of 20 and 79 years, and this number will grow to 629 million by 2045 [51]. The incidence of type 2 diabetes is significantly higher in the elderly than in young people [22].

Skeletal muscle is an important tissue involved in the body’s glucose metabolism (after a mixed meal, the skeletal muscle has important role in glucose disposal) and has an important effect on insulin sensitivity. Skeletal muscle dysfunction is involved in the development of type 2 diabetes. As age increases, many changes and dysfunctions occur in skeletal muscle. Therefore, the increased incidence of type 2 diabetes in the elderly may be closely related to skeletal muscle aging, but the underlying mechanism has not yet been elucidated. This review of the mechanism underlying the increased risk of insulin resistance during the skeletal muscle aging process will provide a more comprehensive explanation for the increased incidence of type 2 diabetes in elderly individuals and will also provide a more comprehensive perspective for the prevention and treatment of type 2 diabetes in elderly populations.

## Aging skeletal muscle is an independent risk factor for insulin resistance

The risk of type 2 diabetes increases with age. Studies have shown that the prevalence of diabetes, impaired fasting glucose or impaired glucose tolerance in 20 to 39-year-olds in the United States was 20.9%, whereas the prevalence in individuals from 40 to 59 years old was 46.9%, the prevalence in individuals from 40 to 59 years old was 67.4%, and the prevalence in individuals ≥ 75 years old was 75.6% [22]. In addition, with increasing age, insulin sensitivity gradually decreased, the body’s glucose regulation ability decreased, and muscle atrophied [47]. Studies have shown that, compared with younger males (30 years old), older males (65–70 years) have reduced glucose metabolism and decreased expression of skeletal muscle glucose transporter 4 (GLUT4) [70]. Older people also exhibit lower skeletal muscle insulin-stimulated Akt activity [59], impaired insulin signaling, and decreased skeletal muscle insulin sensitivity. In addition, skeletal muscle insulin sensitivity in aged mice is reduced, and insulin resistance occurs [24, 84]. These data indicate that the glucose metabolism capacity of the skeletal muscle decreased and the risk of insulin resistance increased. The underlying mechanism is related to a series of changes in skeletal muscle during the aging process.

## The pathophysiological changes in aging skeletal muscle

Skeletal muscle aging refers to the inevitable deterioration of skeletal muscle cell structure and biological function with age increased [23]. Major features such as decreased mitochondrial function, increased intramyocellular lipid, increased inflammatory levels, increased oxidative and endoplasmic reticulum stress, weakened enzyme activities, decreased autophagic capacity, decreased muscle mass and over-activated renin-angiotensin system will appear. These factors can lead to an increased risk of insulin resistance and will be discussed in details.

## Mitochondrial dysfunction

### Mitochondrial dysfunction during skeletal muscle aging

Changes in skeletal muscle mitochondrial structure and function are important features of skeletal muscle aging. In terms of the morphological structure, there is an imbalance in mitochondrial fusion and fission during skeletal muscle aging; mitochondria are larger and rounder, mitochondrial density is reduced, the number of mitochondria is decreased, and the mitochondria are characterized by matrix vacuolation and shorter cristae. Functionally, the ability to synthesize mitochondria and the oxidative capacity are significantly reduced [63], the production of mitochondrial reactive oxygen species (ROS) is increased, the antioxidant capacity is decreased, and the autophagy ability is correspondingly reduced in aging skeletal muscle. Studies have shown that the maximum oxygen uptake of skeletal muscle [85] and the steady-state oxygen uptake [80] decrease with age. Additionally, the maximum ATP production rate and the steady-state ATP production rate of aging skeletal muscle are also reduced [86], and this reduction is more notable in the slow muscle fibers [9]. In addition, the mitochondrial protein synthesis rate in aging skeletal muscle decreased by 40% [82], and mitochondrial protein homeostasis was impaired. These data suggest that a series of changes during skeletal muscle aging cause mitochondrial dysfunction.

### Mitochondrial dysfunction can increase the risk of insulin resistance during skeletal muscle aging

Mitochondria play an important role in skeletal muscle insulin signaling [18], and the normal structure and function of mitochondria are also closely related to skeletal muscle insulin sensitivity. Studies have shown that there is a positive correlation between the number of skeletal muscle mitochondria and insulin sensitivity in elderly individuals [21]. Mitochondria are the major site of ROS production [25], and mitochondrial dysfunction will cause an increase in ROS production, and high levels of ROS impair insulin signaling pathways and induce skeletal muscle insulin resistance [18]. Moreover, due to the decreased oxidative phosphorylation and β-oxidation abilities of aging skeletal muscle, increased intramyocellular lipid accumulation also increases the risk of skeletal muscle insulin resistance. In addition, insulin is critical in maintaining normal mitochondrial function. Insulin inhibits FOXO1 activity, thereby maintaining the integrity of the mitochondrial electron transport chain (METC) and the ratio of NAD +/NADH [18] and protecting mitochondrial function. Therefore, insulin and mitochondria are interdependent; mitochondria require insulin for normal function, mitochondria are required for insulin signaling [99], and skeletal structure and function disorders in aging skeletal muscle increase the risk of insulin resistance.

## Intramyocellular lipid accumulation

### Intramyocellular lipid accumulation increases in aging skeletal muscle

With increasing age, the intramyocellular lipid (IMCL) content also increases gradually [27]. Studies have shown that older men have higher IMCL levels than younger individuals [89]. Moreover, skeletal muscle in older mice also contains higher levels of ceramide (CER) and diacylglycerol (DAG). Compared with young mice, the CER content of skeletal muscle in elderly mice increased two fold, and the DAG content also increased significantly [81]. These data indicate that IMCL accumulation increases in aging skeletal muscle. There are many mechanisms that cause IMCL accumulation. An important mechanism is the decrease in the oxidative phosphorylation and β-oxidation abilities of senile skeletal muscle mitochondria, which leads to a reduction in the oxidative decomposition of lipids in the skeletal muscle.

### Intramyocellular lipid accumulation can increase the risk of insulin resistance in aging skeletal muscle

The accumulation of IMCL can impair skeletal muscle insulin signaling and promote skeletal muscle insulin resistance. Studies have shown that lipid infusion can lead to reduced insulin-induced skeletal muscle protein synthesis and decreased insulin sensitivity in healthy humans [33]. Reducing IMCL levels can improve skeletal muscle insulin sensitivity in obese mice [45]. However, the accumulation of simple triglycerides alone does not lead to insulin resistance [95]. As lipids accumulate, the levels of the CER and DAG increase, which impairs the insulin signaling pathway and promotes insulin resistance [96]. Studies have shown that increased CER levels in skeletal muscle in older individuals can promote insulin resistance [14], while reducing CER and DAG synthesis can improve skeletal muscle insulin sensitivity [61].

In addition, the accumulation of IMCL can also promote the inflammatory pathway [44], thereby promoting skeletal muscle insulin resistance. Studies have shown that skeletal muscle IMCL accumulation can increase the levels of inflammatory factors such as tumor necrosis factor-α (TNF-α), Toll-like receptor 2 (TLR2) and interleukin-1β (IL-1β) [71], thereby promoting the inflammatory pathway. Moreover, the expression of TLR2, TNF-α and IL-1β in old mice is also increased [81], thereby inhibiting Akt and mTOR activity and promoting insulin resistance [62]. Therefore, IMCL accumulation increases the risk of insulin resistance in aging skeletal muscle by impairing the insulin signaling pathway or promoting the inflammatory pathway.

## Inflammation

### Inflammation increases in aging skeletal muscle

The level of inflammation increases during skeletal muscle aging [41]. Studies have shown that, compared with young mice, the expression levels of the skeletal muscle inflammatory markers TLR2, TNF-α and IL-1β are elevated and the level of inflammation is increased in older mice [81]. There are many mechanisms by which the level of inflammation increases in aging skeletal muscle. For example, mitochondrial dysfunction, increased ROS production, IMCL accumulation, and ER stress caused by skeletal muscle aging can promote skeletal muscle inflammation.

### Inflammation increases the risk of insulin resistance in aging skeletal muscle

Inflammation is closely related to skeletal muscle insulin resistance, and increased inflammation promotes skeletal muscle insulin resistance [38]. Studies have shown that TNF-α, monocyte chemoattractant protein-1 (MCP-1), C-reactive protein (CRP), ILs and other inflammatory factors can promote skeletal muscle insulin resistance [78]. After knocking out animal inflammatory factor-related receptors such as TNF-α receptors, skeletal muscle insulin resistance was improved [97]. These data suggest that inflammation promotes skeletal muscle insulin resistance.

Inflammation primarily impairs insulin signaling by activating the IKKβ/NF-κB and JNK pathways, thereby promoting skeletal muscle insulin resistance [26]. Studies have shown that IL-1 can activate the skeletal muscle IKKβ/NF-κB pathway, reduce IRS-1 activity, and promote skeletal muscle insulin resistance [93]. Skeletal muscle insulin resistance improves after the IKKβ/NF-κB pathway is inhibited [48]. Moreover, IL-6 also promotes the expression of cytokine signaling inhibitor 1 (SOCS1) and SOCS3, thereby degrading IRS-1 [93] and promoting insulin resistance. The JNK pathway and TNF-α impair insulin signaling by inducing IRS-1 serine phosphorylation [26]. In addition, inflammation can also promote the release of nitric oxide, thereby inhibiting the PI3K-Akt pathway and promoting insulin resistance [26]. Therefore, inflammation impairs insulin signaling by activating inflammation-related factors as well as the IKKβ/NF-κB and JNK pathways, thereby increasing the risk of insulin resistance in aging skeletal muscle.

## Oxidative stress

### Oxidative stress levels increase in aging skeletal muscle

Mitochondria are the main source of skeletal muscle ROS and are involved in the regulation of various physiological functions of skeletal muscle. At normal concentrations, ROS activate mitogen-activated protein kinase (MAPK) and play an important role in redox signaling and normal cell activity. High levels of ROS can damage mtDNA, proteins and lipids, stimulate apoptosis; and induce skeletal muscle oxidative stress and dysfunction [53]. Studies have shown that as the production of skeletal muscle ROS increases, oxidative stress levels increase, and damage skeletal muscle mtDNA, leading to skeletal muscle mitochondrial dysfunction [53]. After treating mouse skeletal muscle cells with H_2_O_2_, mitochondrial fragmentation increased and respiratory capacity decreased [40]. In addition, increased skeletal muscle ROS production in elderly individuals can directly affect the ATP synthase involved in the ETC pathway, thereby inhibiting ATP production and further reducing skeletal muscle mitochondrial function [98].

There are many reasons for the increase in ROS; the decreased cellular antioxidant capacity caused by decreased antioxidant enzyme (superoxide dismutase, catalase and glutathione peroxidase) activity [15] is an important factor leading to increased ROS production. Studies have shown that the activity of antioxidant enzymes is gradually reduced during the process of skeletal muscle aging [15]. The overexpression of skeletal muscle catalase in aged mice can improve age-related mitochondrial oxidative stress and dysfunction, and enhance mitochondrial energy metabolism [4]. These data suggest that the activity of antioxidant enzymes during skeletal muscle aging is reduced, which leads to an increase in ROS levels, leading to oxidative stress and dysfunction.

### Oxidative stress increases the risk of insulin resistance in aging skeletal muscle

Oxidative stress can increase the risk of insulin resistance in aging skeletal muscle. Biomarkers of skeletal muscle oxidative damage such as malondialdehyde (MDA), protein carbonyl, 4-hydroxy-2-nonenal, hydroperoxide, protein oxidation products, 3-nitrotyrosine, advanced glycosylation products (AGEs), carbohydrate metabolites, and 8-hydroxy-2′-deoxyguanosine reduce skeletal muscle insulin sensitivity and increase the risk of insulin resistance [3, 35]. Studies have shown that after artificially increasing ROS production in myotubes, IRS-1 tyrosine phosphorylation, Akt activation, and GLUT4 translocation to the plasma membrane are impaired [103]. After treatment with losartan, insulin-stimulated IRS-1 phosphorylation, Akt activation, and GLUT4 translocation could be restored [103]. In addition, after treating the soleus muscle with nitric oxide, insulin-stimulated glucose uptake and glycogen synthesis were reduced and the phosphorylation of IRS-1 and Akt was also reduced [43]. These data suggest that increased ROS production in senile skeletal muscle can reduce insulin sensitivity.

Studies of the underlying mechanisms have shown that skeletal muscle oxidative stress can impair insulin signaling and induce insulin resistance [46]. Skeletal muscle oxidative stress activates several serine-threonine kinase pathways such as IKKβ/NF-κB and JNK, leading to IRS-1 degradation [30] and the inhibition of insulin signaling pathways. Moreover, oxidative stress can also inhibit the translocation of GLUT4 to the plasma membrane [49], further reducing the effect of insulin. In addition, oxidative stress can also induce insulin resistance by impairing mitochondrial function. As mentioned earlier, mitochondrial dysfunction can cause a decrease in mitochondrial β-oxidative capacity, leading to IMCL accumulation, inhibiting the activity of PI_3_K, Akt, and GLUT4; and inducing skeletal muscle insulin resistance. However, ROS can damage skeletal muscle mitochondrial function. Therefore, the increase in oxidative stress levels in senile skeletal muscle can activate the IKKβ/NF-κB and JNK pathways and impair mitochondrial function, thereby impairing the insulin signaling pathway in skeletal muscle and increasing the risk for insulin resistance.

## Changes in the activity of enzymes that regulate insulin sensitivity

Protein tyrosine phosphatase 1B (PTP1B) is an enzyme that regulates insulin-sensitivity. PTP1B phosphorylates IRS-1 tyrosine residues, thereby impairing insulin signaling [102] and inducing skeletal muscle insulin resistance. Studies have shown that PTP1B knockout animal models exhibit increased skeletal muscle insulin sensitivity and reduced insulin resistance [60]. However, PTP1B overexpression can promote insulin resistance [29].

The expression level of PTP1B is increased in senile skeletal muscle. Studies have shown that skeletal muscle PTP1B levels are higher and IRS-1 activity is lower in old males (58 years old) than in young males (24 years old) [37]. In addition, PTP1B levels in skeletal muscle were higher, PTP1B interacted with IRS-1 more and insulin resistance was more severe in 28-week-old rats than in 10-week-old rats [5]. These data indicate increased expression of PTP1B in aging skeletal muscle. Therefore, PTP1B increases the risk of insulin resistance in aging skeletal muscle.

## ER stress

### ER stress levels increases in aging skeletal muscle

The endoplasmic reticulum (ER) is an important organelle of eukaryotic cells that is involved in synthesizing, folding, packaging and transporting proteins. During skeletal muscle aging, ER stress levels increase. Studies have shown that ER stress-related factors and markers (GRP78 and CHOP) in the soleus muscles of 32-month-old rats are significantly upregulated compared with the levels in the soleus muscles of 6-month-old rats [77]. Moreover, the expression of ER stress-related factors and markers (GRP78, PDI and CHOP) in the gastrocnemius muscles of 24-month-old mice was also significantly higher than that in 6-month-old mice [50]. These data indicate an increase in ER stress levels in senile skeletal muscle. Studies on the underlying mechanism have shown that ER function declines during skeletal muscle aging, leading to the accumulation of unfolded or misfolded proteins [11], thereby inducing ER stress. In addition, a high level of mitochondrial ROS can also induce ER stress [66], and the skeletal muscle aging process can produce a large amount of ROS, thereby further promoting ER stress.

### ER stress increases the risk of insulin resistance in aging skeletal muscle

ER stress can disrupt protein folding, leading to the accumulation of misfolded protein [8], which can easily induce inflammation and lipid accumulation, thereby impairing insulin signaling and inducing skeletal muscle insulin resistance [69]. Studies have shown that ER stress can reduce the phosphorylation of IRS-1 and Akt, decrease the expression of oxygen-regulated protein 150 (ORP150), which prevents ER stress; and induce insulin resistance [104]. These data suggest that ER stress reduces skeletal muscle insulin sensitivity and induces skeletal muscle insulin resistance [83].

ER stress also promotes skeletal muscle insulin resistance through the JNK pathway. Studies have shown that ER stress activates JNK, thereby phosphorylating IRS-1 serine 307, impairing insulin signaling and inhibiting Akt phosphorylation. As a result, skeletal muscle insulin resistance is promoted [87]. The use of JNK inhibitors reversed the ER stress-induced inhibition of Akt phosphorylation, thereby improving skeletal muscle insulin sensitivity [92]. Therefore, ER stress can increase the risk of insulin resistance in aging skeletal muscle by directly impairing insulin signaling or activating the JNK pathway.

## Autophagy

### Autophagic ability decreases during skeletal muscle aging

The autophagic ability of skeletal muscle gradually decreases with age. Studies have shown that the levels of the p62, LC3-II and LC3-I autophagy markers in the skeletal muscle of aged rats are elevated, indicating that the autophagic ability of the skeletal muscle is weakened, resulting in impaired skeletal muscle function, which is more obvious with age [7]. In addition, the proteolytic capacity of mouse skeletal muscle [100] and rat skeletal muscle [31] also decreased with age, which may be related to the decreased lysosomal protease activity. Studies have found skeletal muscle lysosomal lipid accumulation in senile rats, which results in impaired lysosomal function [76], l decreased lysosomal protease activity [7], and decreased skeletal muscle autophagic ability. These data indicate that the activation of the autophagy-lysosomal pathway is reduced during skeletal muscle aging, which results in decreased autophagy in senile skeletal muscle.

### Decreased autophagy increases the risk of insulin resistance during the skeletal muscle aging process

As mentioned earlier, skeletal muscle oxidative damage increases with age. The autophagy-lysosomal pathway degrades large amounts of skeletal muscle protein, thereby reducing the oxidative damage to the skeletal muscle [58]. Therefore, the decline in skeletal muscle autophagy is not conducive to the prevention of oxidative damage and is closely related to skeletal muscle insulin resistance. Studies have shown that autophagy markers, p62 levels and LC3-II and LC3-I ratios, are significantly increased in insulin-resistant myocytes, and the myocyte autophagic ability is reduced [17]. The expression of skeletal muscle autophagy-related genes (ATG14, RB1CC1/FIP200, GABARAPL1, SQSTM1/p62 and WIPI1) and proteins (LC3BII, SQSTM1/p62 and ATG5) were also significantly decreased in type 2 diabetic patients [72], and skeletal muscle autophagy was decreased. In addition, the insulin-stimulated p-Akt Ser473 levels were decreased and insulin sensitivity was reduced after blocking the autophagy of C2C12 myotubes with the lysosomal inhibitor chloroquine (CLQ) [17]. Insulin resistance was improved after increasing the autophagic ability of the C2C12 myotubes [17] and the L6 myocytes [1]. Therefore, reduced autophagy can increase the risk of insulin resistance during the process of skeletal muscle aging.

## Sarcopenia

### Older people are prone to sarcopenia

Mainly aging-associated skeletal muscle alternations are muscle atrophy, often accompanied by sarcopenia [36]. Sarcopenia is an age-related progressive decline in skeletal muscle mass and function in the absence of other diseases. A progressive decline in muscle mass begins around the age of 40, and muscle mass declines by approximately 8% every decade. After the age of 70, muscle mass decreases by 15% every decade [57]. Additionally, by the age of 70, the cross-sectional area of skeletal muscle is approximately 30% smaller than it was at 20 years old [73]. Additionally, as age increases, the composition of the skeletal muscle fiber types also changes. The proportion of type II muscle fibers is reduced [32], resulting in the muscle mass of type II muscle fibers becoming lower than that of type I muscle fibers. In addition, motor neurons also change. Due to the decreased number and vitality of senile skeletal muscle motor units [54], the neuromuscular dominance is also weakened, which is coupled with the decline in muscle mass in the aging skeletal muscles, resulting in a significant decrease in muscle strength [10]. Before the age of 70, leg strength is reduced by 10–15% every decade. After the age of 70, leg strength is reduced by 25–40% every decade [57]. By the age of 70, skeletal muscle strength is 20–40% lower than that of young people [79]. In addition, studies on the underlying mechanisms have shown that myostatin is a major regulator of skeletal muscle size and mass and is expressed almost exclusively in skeletal muscle [16]. The overexpression of myostatin can cause muscle atrophy and plays an important role in sarcopenia [91]. These data indicate a progressive decline in muscle mass and strength during skeletal muscle aging, which is associated with myostatin.

### Sarcopenia increases the risk of insulin resistance in aging skeletal muscle

Skeletal muscle mass is an important factor in glucose and energy homeostasis [101] and is positively correlated with skeletal muscle insulin sensitivity. Studies have shown that increased muscle mass increases skeletal muscle glucose uptake and improves insulin sensitivity [20]. Sarcopenia can cause skeletal muscle mass and strength to decrease, thereby reducing skeletal muscle insulin sensitivity. Myostatin plays an important role in this process. Studies have shown that elderly mice treated with myostatin inhibitors for 4 weeks exhibited improvements in sarcopenia and increased skeletal muscle insulin sensitivity [16]. Skeletal muscle glucose utilization and insulin sensitivity are also increased in myostatin knockout mice [94]. Therefore, the decreased skeletal muscle mass and strength caused by sarcopenia can increase the risk of insulin resistance in aging skeletal muscle.

## The renin-angiotensin system

### RAS is activated in aging skeletal muscle

The renin-angiotensin system (RAS) plays pleiotropic roles in regulating mammalian pathophysiology. Angiotensin II (Ang II) is a key molecule of RAS and is produced as a result of sequential cleavage of angiotensinogen by renin and angiotensin-converting enzyme (ACE) [56]. RAS has two axes, one is the ACE/ANG II/AT_1_ receptor classic axis. Ang II can bind to the Ang II type 1 (AT_1_) receptor, thereby activating the AT_1_ receptor [2], and leading to cell proliferation, hypertrophic responses, apoptosis, generation of ROS, and tissue inflammation [56]. The other is the ACE2/ANG (1–7)/Mas receptor non-classical axis. Ang II is cleaved by ACE2 to form another peptide Ang (1–7). This ACE2-Ang (1–7) axis, acting via another G protein-coupled receptor Mas, is involved in vasodilatory, anti-fibrotic, and anti-inflammatory properties [52]. These two axes show different changes in aging skeletal muscle. Studies have shown that the skeletal muscle aging process can activate RAS classic axis and activate the AT_1_ receptor [55, 56, 64], which induces inflammation and oxidative stress. However, inhibiting the classic axis can prolong the physiological aging process and promotes longevity in rodents [12]. In addition, RAS non-classical axis weakens in aged skeletal muscles [75]. However, activating the RAS non-classical axis can reduce the aging phenotype in aged mice [75]. These data indicate that the RAS classical axis is activated and the RAS non-classical axis is weakened in aging skeletal muscle.

### RAS activation increases the risk of insulin resistance in aging skeletal muscle

Excessive activation of RAS is closely related to skeletal muscle insulin resistance. Skeletal muscle ACE/ANG II/AT_1_ receptor axis activation can promote skeletal muscle insulin resistance and muscle atrophy [13, 34]. Studies have shown that after injecting Ang II into rats, skeletal muscle glucose tolerance and insulin signaling pathway are impaired, and skeletal muscle insulin resistance appears [90]. After inhibiting the ACE/ANG II/AT_1_ receptor axis, skeletal muscle glucose tolerance and insulin resistance were improved [42]. In addition, the ACE2/ANG (1–7)/Mas receptor axis can inhibit the ACE/ANG II/AT_1_ receptor axis, activate the insulin signaling pathway, and thereby improve skeletal muscle insulin resistance [42]. Studies have shown that after ACE inhibition, skeletal muscle insulin sensitivity is enhanced, and after the Mas receptor is inhibited, the enhancement effect is eliminated [28]. These data indicate that activation of the RAS classical axis can promote skeletal muscle insulin resistance, while activation of the RAS non-classical axis can inhibit the classical axis, thereby improving skeletal muscle insulin resistance. Therefore, activation of the RAS classical axis and weakening of the RAS non-classical axis in aging skeletal muscle may increase the risk of skeletal muscle insulin resistance.

## Integrating these mechanisms

There are also interactions between these mechanisms. Among them, The ER and mitochondria join together at multiple contact sites to form specific domains, termed mitochondria-ER associated membranes (MAMs) [6, 19, 68]. It is closely related to the autophagy process. There are several important autophagy-related proteins in mitochondria, such as ATG5, which is critical for autophagosome formation, translocates to the MAM compartment during phagophore biogenesis and then dissociates from MAMs upon completion of the autophagosome [39]. Therefore, MAM plays an important role in autophagy, while mitochondrial dysfunction and ER stress can decrease autophagy capacity. In addition, increased ROS is an important factor inducing inflammation, while mitochondria and ER are important sources of ROS [67]. Therefore, mitochondrial dysfunction and ER stress can generate a large amount of ROS and induce inflammation and oxidative stress.

In summary, mitochondrial dysfunction and ER stress can decrease autophagy capacity, increased ROS production and IMCL accumulation, and then induce inflammation and oxidative stress. Furthermore, the over-activated renin-angiotensin system also increases inflammation levels and induces oxidative stress. In addition, the occurrence of sarcopenia will exacerbate the above processes. Finally, increased inflammation and oxidative stress can impair mitochondrial function and exacerbate ER stress in turn, thereby further exacerbating the above processes and increasing the risk of insulin resistance.

## Prevention and treatment

The aforementioned mechanisms, such as mitochondrial oxidative ability, inflammation, oxidative stress, insulin sensitivity regulating enzymes, ER stress, autophagy ability, and RAS axis, can be used as targets for the prevention and treatment of aging skeletal muscle insulin resistance. In addition, there are non-pharmacological treatments, such as exercise, that can prevent and treat insulin resistance in aging skeletal muscle. Studies have shown that exercise can increase skeletal muscle mass and improve skeletal muscle insulin sensitivity [36, 65, 88]. Exercise can also enhance mitochondrial oxidative capacity, enhance skeletal muscle autophagy and antioxidant capacity, reduce oxidative stress and inflammation levels [36, 74], and improve skeletal muscle insulin resistance. Therefore, both pharmacological treatments targeting these mechanisms and exercise can prevent and treat aging skeletal muscle insulin resistance.

## Conclusions

**Fig. 1:**
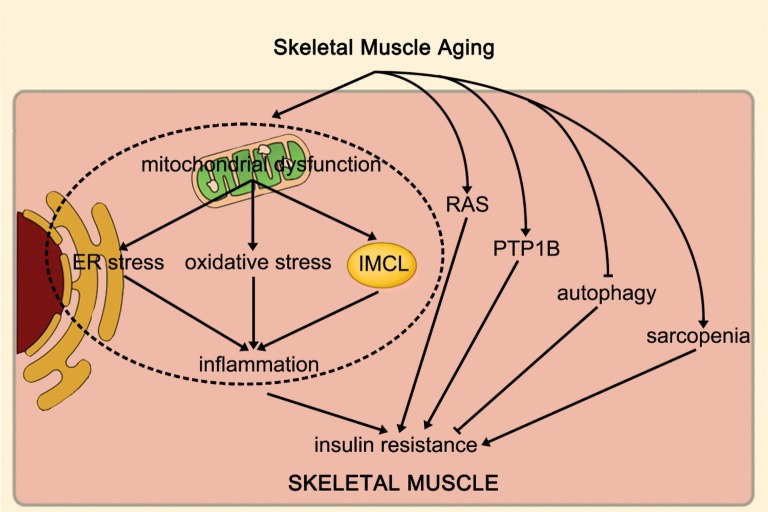
Skeletal muscle aging can increase insulin resistance by promoting mitochondrial dysfunction, IMCL accumulation, inflammation, oxidative stress, PTP1B expression, ER stress, decreased autophagy, sarcopenia and over-activated RAS. In addition, skeletal muscle mitochondrial dysfunction promotes IMCL accumulation and induces oxidative stress and ER stress, moreover, IMCL accumulation, oxidative stress, and ER stress can induce inflammation. *IMCL* intramyocellular lipid, *PTP1B* protein tyrosine phosphatase 1B, *ER* endoplasmic reticulum, *RAS* renin-angiotensin system

An increased risk of senile skeletal muscle insulin resistance is associated with skeletal muscle dysfunction. During the aging of skeletal muscle, mitochondrial dysfunction, intramyocellular lipid accumulation, increased inflammation, oxidative stress, changes in the activities of enzymes that regulate insulin sensitivity, endoplasmic reticulum stress, decreased autophagy, sarcopenia and over-activated RAS all induce skeletal muscle insulin resistance. These processes can impair skeletal muscle insulin sensitivity and increase the risk of insulin resistance and type 2 diabetes during the skeletal muscle aging process (Fig. 1). Of course, pharmacological treatments targeting these mechanisms and exercise can prevent and treat aging skeletal muscle insulin resistance. Therefore, in view of the above-mentioned aspects closely related to aging skeletal muscle insulin resistance, further exploration of relevant mechanisms and development of related drugs require further research in the future.

## Data Availability

All data generated or analysed during this study are included in this published article (Fig. 1).

## Abbreviations

IDF: International diabetes federation
GLUT4: Glucose transporter 4
ROS: Reactive oxygen species
METC: Mitochondrial electron transport chain
IMCL: Intramyocellular lipid
CER: Ceramide
DAG: Diacylglycerol
TNF-α: Tumor necrosis factor-α
TLR2: Toll-like receptor 2
IL-1β: Interleukin-1β
MCP-1: Monocyte chemoattractant protein-1
CRP: C-reactive protein
SOCS1: Cytokine signaling inhibitor 1
MAPK: Mitogen-activated protein kinase
AGEs: Advanced glycosylation products
PTP1B: Protein tyrosine phosphatase 1B
ER: Endoplasmic reticulum
ORP150: Oxygen-regulated protein 150
RAS: Renin-angiotensin system
ACE: Angiotensin-converting enzyme
Ang II: Angiotensin II
